# Crystal structure of bis­(4-acetyl­anilinium) tetra­chlorido­mercurate(II)

**DOI:** 10.1107/S2056989015022355

**Published:** 2015-11-28

**Authors:** Manickam Thairiyaraja, Arumugam Elangovan, Ganesh Arivazhagan, Kuthambalam Selvaraju, Subbiah Thamotharan

**Affiliations:** a PG & Research Department of Physics, Government Arts College, Ariyalur 621 713, India; b Department of Chemistry, Thiagarajar College, Madurai 625 009, India; c Department of Physics, Thiagarajar College, Madurai 625 009, India; d Biomolecular Crystallography Laboratory, Department of Bioinformatics, School of Chemical and Biotechnology, SASTRA University, Thanjavur 613 401, India

**Keywords:** crystal structure, isotypism, mercury(II), hydrogen bonding

## Abstract

The structure of the title salt, (C_8_H_10_NO)_2_[HgCl_4_], is isotypic with that of the cuprate(II) and cobaltate(II) analogues. The asymmetric unit contains one 4-acetyl­anilinium cation and one half of a tetra­chlorido­mercurate(II) anion (point group symmetry *m*). The Hg—Cl distances are in the range 2.4308 (7)–2.5244 (11) Å and the Cl—Hg—Cl angles in the range of 104.66 (2)–122.94 (4)°, indicating a considerable distortion of the tetra­hedral anion. In the crystal, cations are linked by an inter­molecular N—H⋯O hydrogen-bonding inter­action, leading to a *C*(8) chain motif with the chains extending parallel to the *b* axis. There is also a π–π stacking inter­action with a centroid-to-centroid distance of 3.735 (2) Å between neighbouring benzene rings along this direction. The anions lie between the chains and inter­act with the cations through inter­molecular N—H⋯Cl hydrogen bonds, leading to the formation of a three-dimensional network structure.

## Related literature   

For the structures of the isotypic tetra­chlorido­cuprate(II) and tetra­chlorido­cobaltate(II) analogues, see: Elangovan *et al.* (2007 ▸) and Thairiyaraja *et al.* (2015 ▸), respectively.
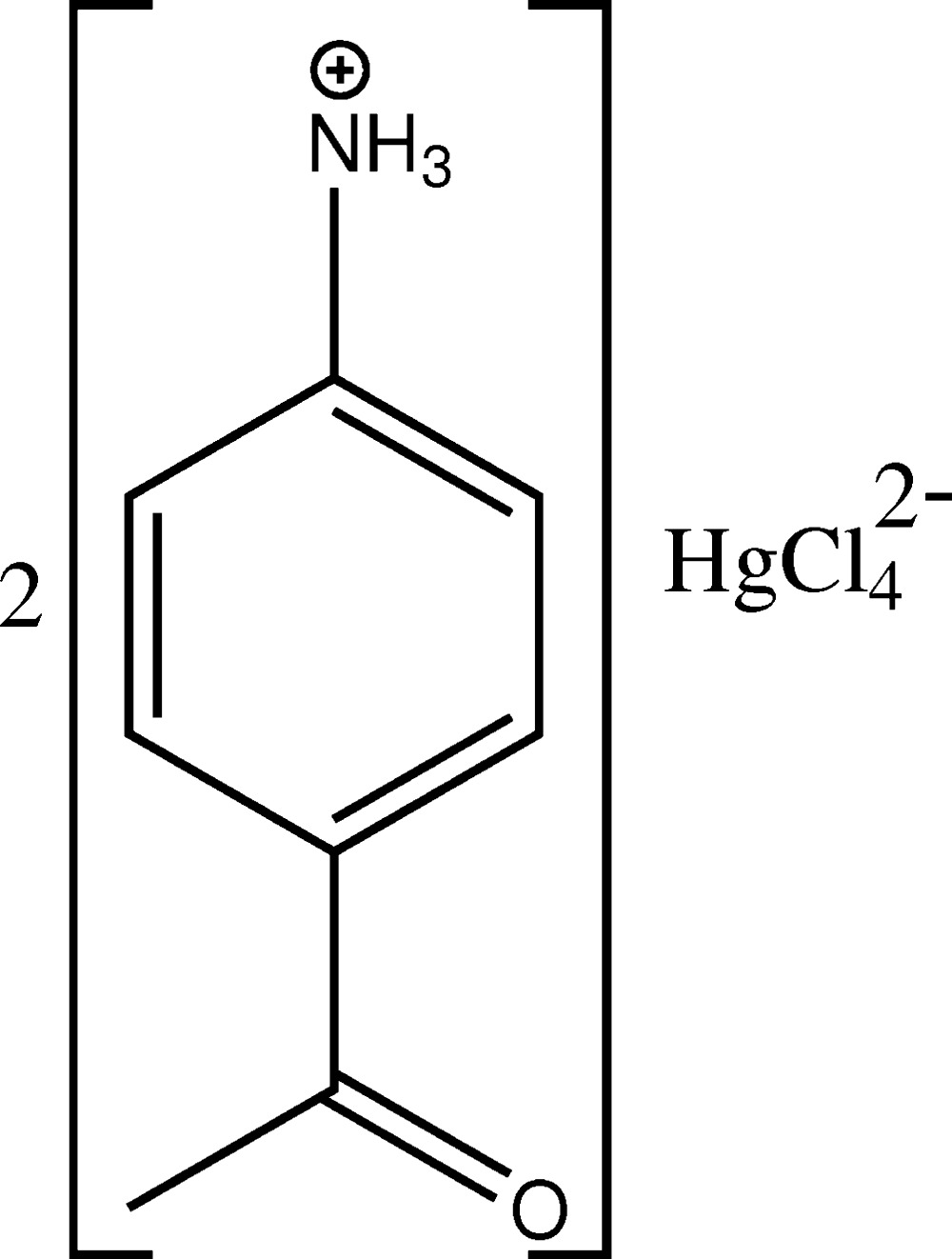


## Experimental   

### Crystal data  

(C_8_H_10_NO)_2_[HgCl_4_]
*M* *_r_* = 614.73
Orthorhombic, 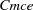

*a* = 19.9231 (6) Å
*b* = 15.3515 (6) Å
*c* = 13.7587 (5) Å
*V* = 4208.1 (3) Å^3^
*Z* = 8
Mo *K*α radiation
μ = 7.84 mm^−1^
*T* = 293 K
0.30 × 0.25 × 0.20 mm

### Data collection  

Bruker SMART APEX CCD diffractometer
Absorption correction: multi-scan (*SADABS*; Bruker, 2004 ▸) *T*_min_ = 0.202, *T*_max_ = 0.303
26289 measured reflections
2988 independent reflections
2152 reflections with *I* > 2σ(*I*)
*R*_int_ = 0.031

### Refinement  

*R*[*F* ^2^ > 2σ(*F* ^2^)] = 0.023
*wR*(*F* ^2^) = 0.056
*S* = 1.04
2988 reflections
131 parameters
3 restraints
H atoms treated by a mixture of independent and constrained refinement
Δρ_max_ = 0.77 e Å^−3^
Δρ_min_ = −0.93 e Å^−3^

### 

Data collection: *APEX2* (Bruker, 2004 ▸); cell refinement: *SAINT* (Bruker, 2004 ▸); data reduction: *SAINT*; program(s) used to solve structure: coorsdinates taken from an isotypic compound; program(s) used to refine structure: *SHELXL2014* (Sheldrick, 2015 ▸); molecular graphics: *PLATON* (Spek, 2009 ▸); software used to prepare material for publication: *publCIF* (Westrip, 2010 ▸).

## Supplementary Material

Crystal structure: contains datablock(s) I, New_Global_Publ_Block. DOI: 10.1107/S2056989015022355/wm5243sup1.cif

**Fig. 1.**
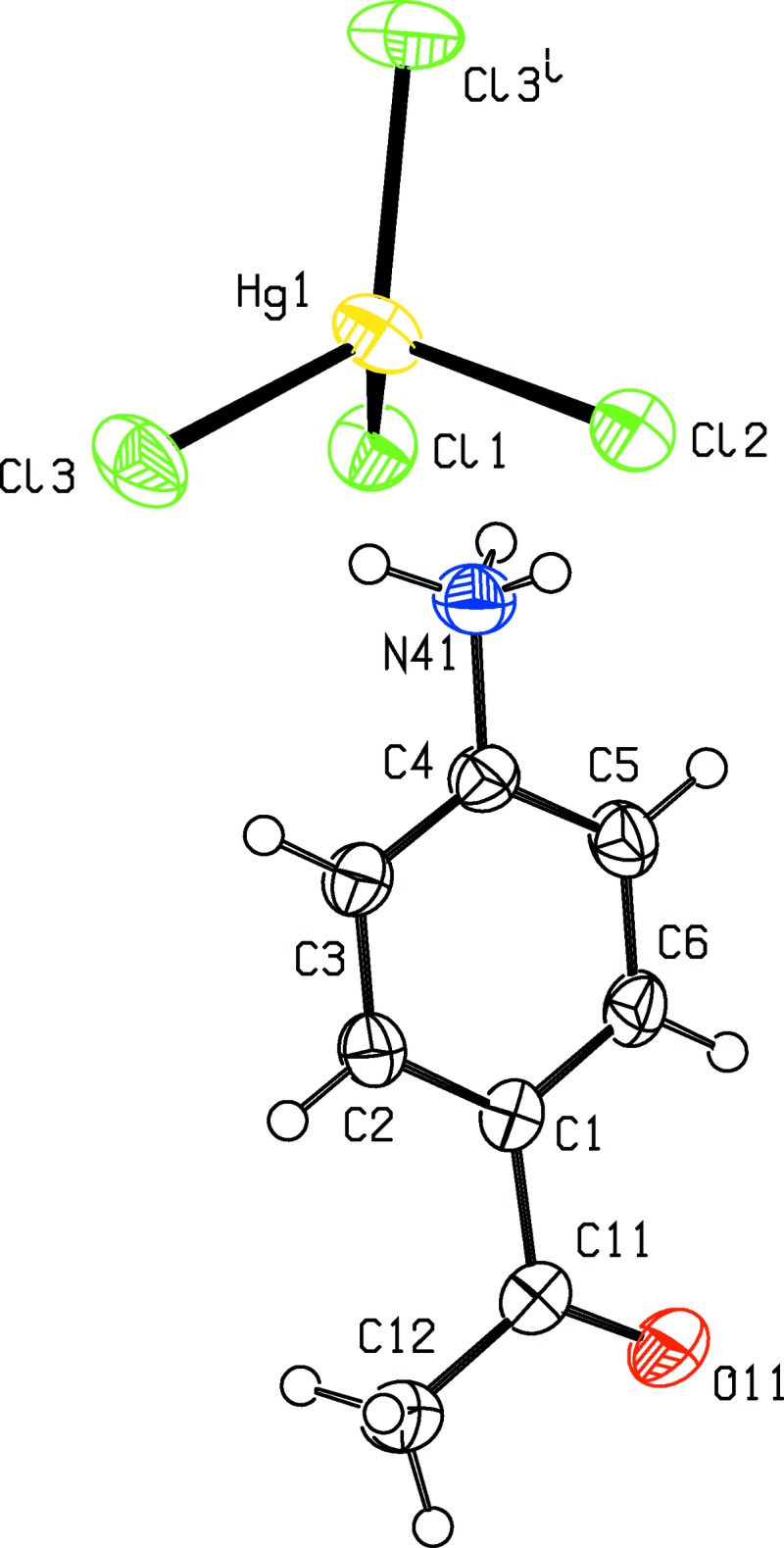
The molecular components of the title salt, showing displacement ellipsoids at the 50% probability level. [Symmetry code: (i) -x, y, z.]

**Fig. 2.**
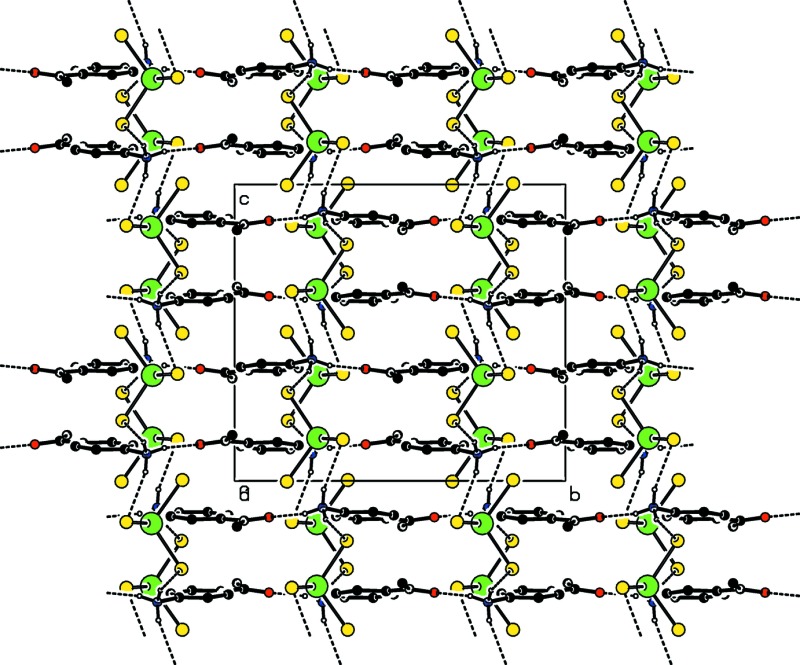
The crystal packing of the title salt viewed along the a axis. Hydrogen bonds are shown as dashed lines; H atoms bound to C were omitted for clarity.

Structure factors: contains datablock(s) I. DOI: 10.1107/S2056989015022355/wm5243Isup2.hkl

x y z. . DOI: 10.1107/S2056989015022355/wm5243fig1.tif
The mol­ecular components of the title salt, showing displacement ellipsoids at the 50% probability level. [Symmetry code: (i) −*x*, *y*, *z.*]

a . DOI: 10.1107/S2056989015022355/wm5243fig2.tif
The crystal packing of the title salt viewed along the *a* axis. Hydrogen bonds are shown as dashed lines; H atoms bound to C were omitted for clarity.

CCDC reference: 1047866

Additional supporting information:  crystallographic information; 3D view; checkCIF report

## Figures and Tables

**Table 1:** Hydrogen-bond geometry (Å, °)

*D*—H⋯*A*	*D*—H	H⋯*A*	*D*⋯*A*	*D*—H⋯*A*
N41—H41*A*⋯O11^i^	0.90 (2)	1.92 (2)	2.792 (3)	162 (4)
N41—H41*C*⋯Cl2^ii^	0.90 (2)	2.34 (2)	3.206 (3)	162 (3)
N41—H41*B*⋯Cl3^iii^	0.89 (2)	2.49 (2)	3.326 (3)	158 (3)

Symmetry codes: (i) 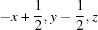; (ii) 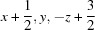; (iii) 
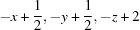
.

## supplementary crystallographic information

### S1. Synthesis and crystallization

A solution of 4-amino­aceto­phenone (20 mmol) in 2 ml of HCl and deionized water (10 ml) was added to a 10 ml solution of HgCl_2_ (10 mmol). The resulting solution was concentrated and kept unperturbed at ambient temperature for crystallization. Single crystals suitable for X-ray diffraction were obtained within a week.

### S2. Refinement

Since the title salt is isotypic with its tetra­chloridocobaltate and tetra­chloridocuprate analogues, it was refined with the coordinates of the tetra­chloridocobaltate salt (Thairiyaraja *et al.*, 2015) as a starting model. The ammonium H atoms were located from a difference Fourier map and were refined with a distance restraint of N—H = 0.89 (2) Å. The methyl H atoms were constrained to an ideal geometry (C—H = 0.96 Å) with *U*_iso_(H) = 1.5*U*_eq_(C), but were allowed to rotate freely about the C—C bond. The remaining H atoms were positioned geometrically and refined using a riding model approximation with C—H = 0.93 Å and *U*_iso_(H) = 1.2*U*_eq_(C). At this stage, the maximum residual electron density of 0.77 e Å^-3^ suggested the presence of a possible atom site at Wyckofff position 4*a* at a distance of 2.88 Å near atom H5. This electron density was assumed to be the O atom of a water molecule and was refined with isotropic displacement parameters. However, the resultant model had higher reliability factors and a very high isotropic atomic displacement parameter for this O atom. As a consequence, this water O atom was not included in the final model.

### Crystal data

**Table:**

(C_8_H_10_NO)_2_[HgCl_4_]	*D*_x_ = 1.941 Mg m^−^^3^
*M**_r_* = 614.73	Mo *K*α radiation, λ = 0.71073 Å
Orthorhombic, *C**m**c**e*	Cell parameters from 9224 reflections
*a* = 19.9231 (6) Å	θ = 2.2–29.3°
*b* = 15.3515 (6) Å	µ = 7.84 mm^−^^1^
*c* = 13.7587 (5) Å	*T* = 293 K
*V* = 4208.1 (3) Å^3^	Block, orange
*Z* = 8	0.30 × 0.25 × 0.20 mm
*F*(000) = 2352	

### Data collection

**Table:**

Bruker SMART APEX CCD diffractometer	2152 reflections with *I* > 2σ(*I*)
Radiation source: fine-focus sealed tube	*R*_int_ = 0.031
ω and φ scan	θ_max_ = 29.4°, θ_min_ = 2.2°
Absorption correction: multi-scan (*SADABS*; Bruker, 2004)	*h* = −27→27
*T*_min_ = 0.202, *T*_max_ = 0.303	*k* = −19→21
26289 measured reflections	*l* = −18→19
2988 independent reflections	

### Refinement

**Table:**

Refinement on *F*^2^	Primary atom site location: isomorphous structure methods
Least-squares matrix: full	Hydrogen site location: mixed
*R*[*F*^2^ > 2σ(*F*^2^)] = 0.023	H atoms treated by a mixture of independent and constrained refinement
*wR*(*F*^2^) = 0.056	*w* = 1/[σ^2^(*F*_o_^2^) + (0.0217*P*)^2^ + 6.8872*P*] where *P* = (*F*_o_^2^ + 2*F*_c_^2^)/3
*S* = 1.04	(Δ/σ)_max_ < 0.001
2988 reflections	Δρ_max_ = 0.77 e Å^−^^3^
131 parameters	Δρ_min_ = −0.93 e Å^−^^3^
3 restraints	

### Special details

**Table:**

Geometry. All e.s.d.'s (except the e.s.d. in the dihedral angle between two l.s. planes) are estimated using the full covariance matrix. The cell e.s.d.'s are taken into account individually in the estimation of e.s.d.'s in distances, angles and torsion angles; correlations between e.s.d.'s in cell parameters are only used when they are defined by crystal symmetry. An approximate (isotropic) treatment of cell e.s.d.'s is used for estimating e.s.d.'s involving l.s. planes.

### Fractional atomic coordinates and isotropic or equivalent isotropic displacement parameters (Å^2^)

**Table:**

	*x*	*y*	*z*	*U*_iso_*/*U*_eq_	
O11	0.18874 (11)	0.60387 (14)	0.87716 (17)	0.0544 (6)	
N41	0.37059 (13)	0.26610 (16)	0.9093 (2)	0.0430 (6)	
H41A	0.359 (2)	0.2125 (16)	0.889 (3)	0.084 (14)*	
H41B	0.3848 (19)	0.270 (2)	0.9703 (16)	0.072 (13)*	
H41C	0.4057 (15)	0.276 (3)	0.870 (2)	0.067 (12)*	
C1	0.22199 (12)	0.45798 (17)	0.87221 (17)	0.0297 (5)	
C2	0.20483 (13)	0.37068 (17)	0.86957 (19)	0.0359 (6)	
H2	0.1603	0.3546	0.8606	0.043*	
C3	0.25367 (13)	0.30686 (17)	0.8803 (2)	0.0373 (6)	
H3	0.2423	0.2481	0.8784	0.045*	
C4	0.31897 (12)	0.33222 (17)	0.89352 (19)	0.0321 (5)	
C5	0.33780 (13)	0.41877 (18)	0.8947 (2)	0.0374 (6)	
H5	0.3825	0.4346	0.9029	0.045*	
C6	0.28863 (13)	0.48110 (18)	0.88350 (19)	0.0349 (6)	
H6	0.3005	0.5397	0.8835	0.042*	
C11	0.17064 (14)	0.52843 (18)	0.86546 (19)	0.0341 (6)	
C12	0.09920 (13)	0.5071 (2)	0.8458 (2)	0.0482 (8)	
H12A	0.0742	0.5600	0.8372	0.072*	
H12B	0.0811	0.4750	0.8996	0.072*	
H12C	0.0962	0.4725	0.7878	0.072*	
Hg1	0.0000	0.24841 (2)	0.85591 (2)	0.04734 (7)	
Cl1	0.0000	0.34199 (7)	1.00252 (8)	0.0479 (2)	
Cl2	0.0000	0.33901 (7)	0.70279 (8)	0.0455 (2)	
Cl3	0.10720 (4)	0.17292 (6)	0.86099 (7)	0.0607 (2)	

### Atomic displacement parameters (Å^2^)

**Table:**

	*U*^11^	*U*^22^	*U*^33^	*U*^12^	*U*^13^	*U*^23^
O11	0.0515 (12)	0.0266 (12)	0.0850 (17)	0.0019 (9)	−0.0071 (11)	−0.0038 (10)
N41	0.0335 (11)	0.0344 (16)	0.061 (2)	0.0014 (9)	−0.0018 (11)	0.0055 (12)
C1	0.0341 (12)	0.0274 (14)	0.0275 (14)	−0.0026 (9)	0.0008 (10)	−0.0008 (10)
C2	0.0309 (12)	0.0321 (15)	0.0448 (16)	−0.0053 (10)	−0.0016 (11)	−0.0005 (11)
C3	0.0361 (13)	0.0236 (14)	0.0523 (18)	−0.0041 (10)	−0.0008 (12)	0.0023 (11)
C4	0.0331 (12)	0.0269 (14)	0.0365 (14)	0.0009 (10)	−0.0009 (10)	0.0030 (11)
C5	0.0326 (12)	0.0351 (16)	0.0445 (16)	−0.0062 (11)	−0.0037 (11)	0.0013 (12)
C6	0.0387 (14)	0.0248 (14)	0.0410 (15)	−0.0054 (10)	−0.0025 (11)	−0.0022 (11)
C11	0.0387 (13)	0.0299 (15)	0.0336 (14)	0.0010 (10)	0.0017 (11)	−0.0014 (11)
C12	0.0359 (13)	0.0407 (17)	0.068 (2)	0.0042 (12)	0.0015 (14)	−0.0065 (15)
Hg1	0.03172 (8)	0.05938 (13)	0.05092 (11)	0.000	0.000	−0.00393 (9)
Cl1	0.0538 (5)	0.0448 (6)	0.0453 (6)	0.000	0.000	−0.0061 (5)
Cl2	0.0420 (5)	0.0490 (6)	0.0455 (6)	0.000	0.000	−0.0018 (5)
Cl3	0.0383 (4)	0.0555 (5)	0.0883 (6)	0.0145 (3)	−0.0098 (4)	−0.0202 (4)

### Geometric parameters (Å, º)

**Table:**

O11—C11	1.224 (3)	C4—C5	1.381 (4)
N41—C4	1.461 (3)	C5—C6	1.378 (4)
N41—H41A	0.901 (19)	C5—H5	0.9300
N41—H41B	0.887 (19)	C6—H6	0.9300
N41—H41C	0.899 (19)	C11—C12	1.486 (4)
C1—C6	1.383 (4)	C12—H12A	0.9600
C1—C2	1.384 (4)	C12—H12B	0.9600
C1—C11	1.492 (4)	C12—H12C	0.9600
C2—C3	1.389 (4)	Hg1—Cl3^i^	2.4308 (7)
C2—H2	0.9300	Hg1—Cl3	2.4308 (7)
C3—C4	1.370 (4)	Hg1—Cl1	2.4764 (11)
C3—H3	0.9300	Hg1—Cl2	2.5244 (11)
			
C4—N41—H41A	114 (3)	C4—C5—H5	120.9
C4—N41—H41B	109 (2)	C5—C6—C1	121.1 (2)
H41A—N41—H41B	116 (3)	C5—C6—H6	119.4
C4—N41—H41C	110 (3)	C1—C6—H6	119.4
H41A—N41—H41C	100 (3)	O11—C11—C12	121.0 (3)
H41B—N41—H41C	108 (3)	O11—C11—C1	118.4 (2)
C6—C1—C2	119.3 (2)	C12—C11—C1	120.6 (2)
C6—C1—C11	118.6 (2)	C11—C12—H12A	109.5
C2—C1—C11	122.1 (2)	C11—C12—H12B	109.5
C1—C2—C3	120.5 (2)	H12A—C12—H12B	109.5
C1—C2—H2	119.8	C11—C12—H12C	109.5
C3—C2—H2	119.8	H12A—C12—H12C	109.5
C4—C3—C2	118.6 (2)	H12B—C12—H12C	109.5
C4—C3—H3	120.7	Cl3^i^—Hg1—Cl3	122.94 (4)
C2—C3—H3	120.7	Cl3^i^—Hg1—Cl1	104.66 (2)
C3—C4—C5	122.2 (2)	Cl3—Hg1—Cl1	104.66 (2)
C3—C4—N41	119.4 (2)	Cl3^i^—Hg1—Cl2	106.66 (3)
C5—C4—N41	118.4 (2)	Cl3—Hg1—Cl2	106.66 (3)
C6—C5—C4	118.3 (2)	Cl1—Hg1—Cl2	111.11 (4)
C6—C5—H5	120.9		
			
C6—C1—C2—C3	1.4 (4)	C4—C5—C6—C1	0.6 (4)
C11—C1—C2—C3	−177.2 (2)	C2—C1—C6—C5	−1.8 (4)
C1—C2—C3—C4	0.1 (4)	C11—C1—C6—C5	176.9 (2)
C2—C3—C4—C5	−1.3 (4)	C6—C1—C11—O11	−5.2 (4)
C2—C3—C4—N41	177.3 (3)	C2—C1—C11—O11	173.4 (3)
C3—C4—C5—C6	0.9 (4)	C6—C1—C11—C12	175.3 (2)
N41—C4—C5—C6	−177.6 (3)	C2—C1—C11—C12	−6.1 (4)

Symmetry code: (i) −*x*, *y*, *z*.

### Hydrogen-bond geometry (Å, º)

**Table:**

*D*—H···*A*	*D*—H	H···*A*	*D*···*A*	*D*—H···*A*
N41—H41*A*···O11^ii^	0.90 (2)	1.92 (2)	2.792 (3)	162 (4)
N41—H41*C*···Cl2^iii^	0.90 (2)	2.34 (2)	3.206 (3)	162 (3)
N41—H41*B*···Cl3^iv^	0.89 (2)	2.49 (2)	3.326 (3)	158 (3)

Symmetry codes: (ii) −*x*+1/2, *y*−1/2, *z*; (iii) *x*+1/2, *y*, −*z*+3/2; (iv) −*x*+1/2, −*y*+1/2, −*z*+2.
