# Supplementary figures and images for: Crystal structure of bis­(4-acetyl­anilinium) tetra­chlorido­mercurate(II)
Source: Acta Crystallogr E Crystallogr Commun. 2015 Nov 28;71(Pt 12):m236–7. doi: 10.1107/S2056989015022355 (PMC4719851; doi:10.1107/S2056989015022355)

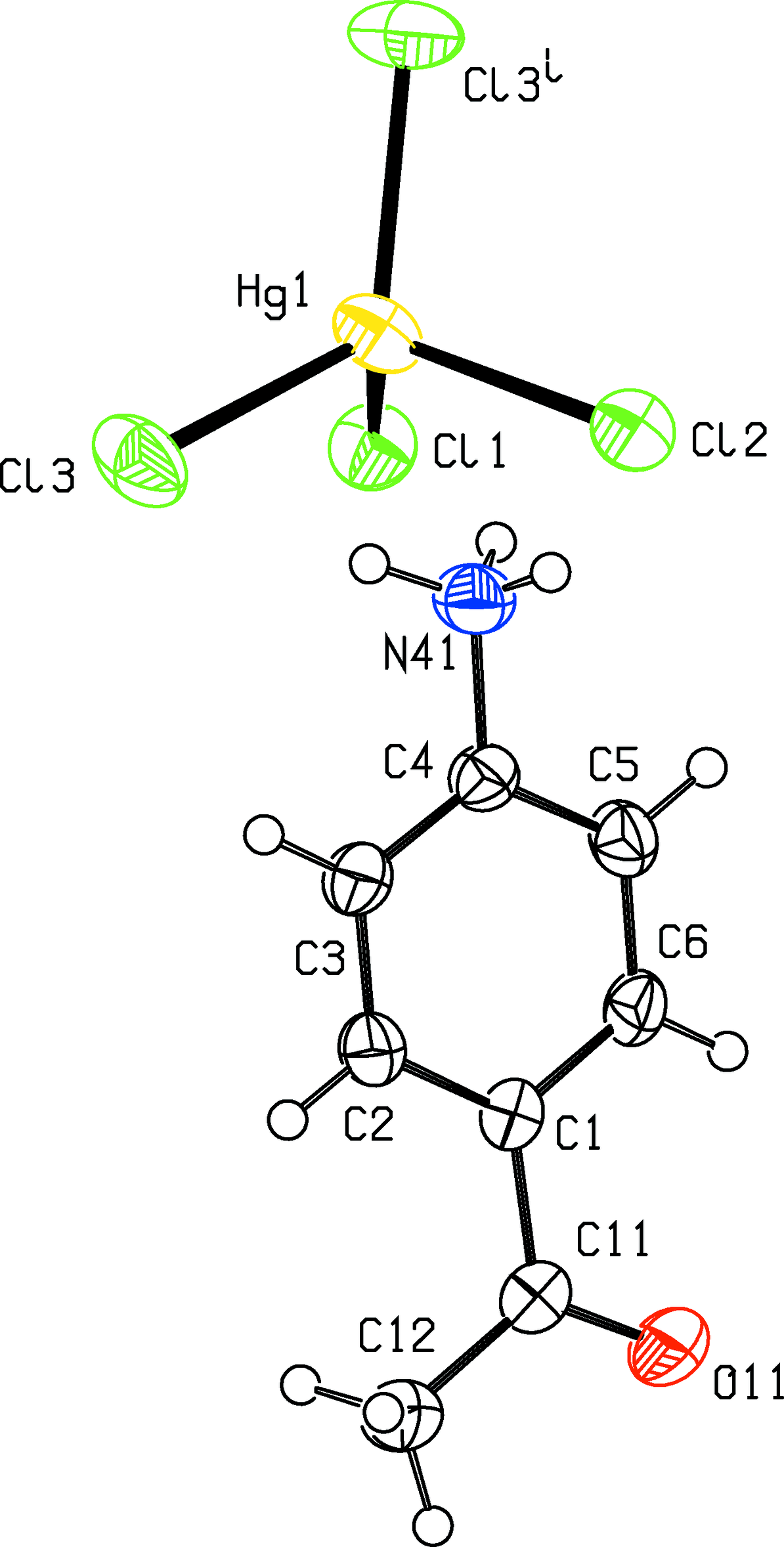

Supplement: Supplementary file 3 [file e-71-0m236-fig1.tif]

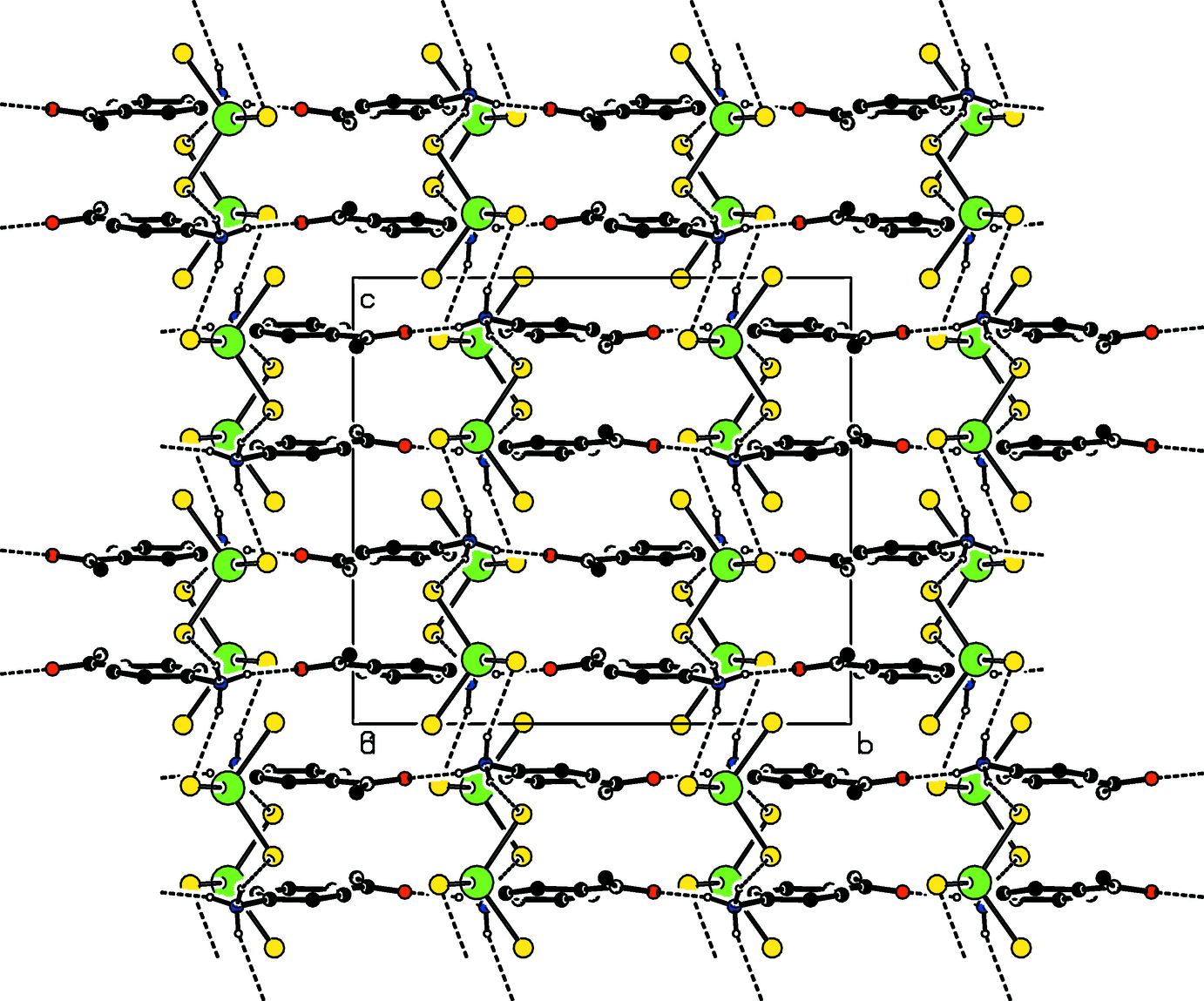

Supplement: Supplementary file 4 [file e-71-0m236-fig2.tif]
